# A modified live bat influenza A virus-based vaccine prototype provides full protection against HPAIV H5N1

**DOI:** 10.1038/s41541-020-0185-6

**Published:** 2020-05-15

**Authors:** Jacob Schön, Wei Ran, Marco Gorka, Martin Schwemmle, Martin Beer, Donata Hoffmann

**Affiliations:** 1 Institute of Diagnostic Virology, Friedrich-Loeffler-Institut, Federal Research Institute for Animal Health, Greifswald-Insel Riems, Germany; 2 Institute of Virology, University Medical Center Freiburg, Freiburg, Germany; 3 Faculty of Medicine, University of Freiburg, Freiburg, Germany

**Keywords:** Influenza virus, Live attenuated vaccines

## Abstract

Highly pathogenic avian influenza viruses (HPAIVs) of subtype H5 are a major threat for poultry holdings worldwide, here especially the zoonotic Asian H5N1 viruses. These HPAIVs have caused more than 500 fatal spillover infections from poultry to humans, with a looming danger of a new pandemic by establishing human-to-human transmissions. Besides culling measures in infected farms in endemic areas, vaccination is the major tool against HPAIV. However, the mainly used inactivated preparations have several limitations, like application to the individual animal by injection and a reduced efficiency. Here we present a modified live influenza vaccine prototype, which is based on the H17N10 bat influenza virus. The new chimeric vaccine strain R65_mono_/H17N10 was able to provide full protection against a lethal challenge infection with HPAIV H5N1 of juvenile and subadult chickens, as well as ferrets after oronasal immunization. In addition, the H5 vaccine prototype cannot reassort with avian influenza viruses and therefore is a promising tool against HPAIV H5 infection, allowing new vaccination strategies for efficient disease control.

## Introduction

Avian influenza (AI) can affect more than 100 different avian species and all types of domestic birds’, and wild waterfowl represents the natural reservoir^1^. The causative agent is influenza A virus (IAV), which is an orthomyxovirus with eight negative-sense single-stranded RNA genome segments, coding for at least 13 proteins^2^. On the basis of the cross-reactivity of antibodies (Ab) specific for the IAV membrane glycoproteins hemagglutinin (H1-18) and neuraminidase (N1-11), the viruses are classified in different subtypes. Owing to their genetic and phylogenetic divergence, the bat-specific H17N10 and H18N11 constitute a special group within the influenza A genus, leading to the term “influenza A-like virus”^3,4^. These differences include an incompatibility of the bat influenza package sequences (PS) with conventional IAVs^5^.

In gallinaceous birds, HPAIVs of the subtypes H5 and H7 can cause a systemic disease with very high mortality rates of up to 100%, with sometimes sudden death as the only recognizable clinical sign. Characteristic for all HPAIVs is a polybasic proteolytic cleavage site in the HA protein^6^. In 1997, the first HPAIV H5N1 virus emerged and, besides poultry, infected humans including fatal cases^7^. The original H5N1 viruses have further diversified over the time, and new viruses have emerged by replacing the N1 encoding gene segment via reassortment with N2, N3, N5, N6, N8, or N9 gene segments^8^. Between January 2013 and August 2018, several HPAIV subtypes caused the loss of 122 million domestic birds globally, with H5N1 having a big share^9^. As HPAIV now is endemic in many countries^10,11^, causing considerable economic damage, the risk of zoonotic spillover events is eminent^12,13^.

In most countries, the main strategy to control AI in poultry relies on prevention by strict biosecurity measures, and in the case of outbreaks, the implementation of restriction areas and depopulation of affected farms^11^. In comparison to the economic losses caused by these measures^9^, effective vaccination could help to prevent disease spread and further zoonotic spillover infections^14^. Countries where HPAIV is endemic like China, Egypt, or Indonesia already have used inactivated influenza vaccines (IIVs) or vector vaccines, mostly in case of emergency response^10^. A recent and successful example is the vaccination campaign against the H7N9 HPAIV which emerged in China in 2017^15,16^. The mass vaccination of poultry in China, using a recombinant bivalent H5/H7 IIV, led to a marked drop in human spillover infections from 1567 cases (615 fatal) in the season 2016/2017 to only three reported cases in the season 2017/2018^14^.

However, there are experiments which indicate that influenza live vaccines can “elicit higher levels of innate responses, mucosal IgA antibodies, and heterologous protection in 1-day-old chickens compared to IIV”^17^. Modified live influenza vaccines (MLIV) are able to induce a broad humoral (systemic and mucosal) and cellular immune response. Mimicking a natural infection and stimulating the immune system more broadly is advantageous for MLIVs^17,18^. The avian IIVs Ab response is mainly based on a systemic IgY, the avian counterpart to mammalian IgG, whereas MLIVs induce also mucosal IgA secretion in the upper respiratory tract contributing to their efficiency^18^. A major advantage of MLIVs is the possibility of oral application, which allows easy mass vaccination as done for other live vaccines, e.g. against Newcastle disease^19,20^. The current MLIVs are based on apathogenic influenza viruses, and attenuation is, e.g. achieved by using truncated NS1 proteins^21^ or restricted replication capacities due to cold-adaptation^22^ or the introduction of elastase-specific cleavage sites into the HA precursor protein^23^. Although cold-adapted MLIVs have been licensed for several years for use in humans, there are no MLIVs licensed for poultry yet, and only two types of vector-based live vaccines are on the market^18^. These vector-based vaccines could induce a broader protection than IIVs, but it is still difficult to express more than the HA protein in the vector backbone, as it is known that also the neuraminidase (NA), the matrix protein (M2), and the nucleoprotein (NP) are beneficial for a broader and more effective immunity^24^. In addition, preexisting immunity to the used vector viruses limits the efficacy of these vaccines^17^. Of course, apathogenicity and efficacy of MLIVs in day-old chicks is an issue^17,25^, mainly due to their immature immune system^26,27^. However, early immunization enhances the chances to prevent an infection. Therefore, vaccination of day-old chicks is a very interesting application, and it has been shown that MLIVs are more effective than IIVs for the immunization of young individuals^17,28^.

A major drawback of MLIV is the risk of reassortment with wild-type IAVs. The World Organization for Animal Health (OIE) does not recommend the use of influenza live vaccines due to the risk of reassortment^29^. There are different research attempts to generate reassortment incompatible vaccines: Chimeric influenza B viruses (IBV) were constructed, which carry the HA segment of IAVs with IBV packaging sequences (PS) and therefore do not reassort with IAVs^30^. These chimeric viruses efficiently protected mice against lethal IAV infection^30^. In a second approach the PS were switched between the HA and the non-structural protein (NS) gene segment; preventing reassortment of these segments^31^. None of these approaches has been tested in poultry or ferrets so far. Chickens are used by default for pathogenicity and vaccine efficacy studies for poultry^32^. Ferrets are the mammalian counterpart, used for vaccine safety evaluation^33^.

The bat-originated H17N10^34^ shows an incompatibility of the bat influenza PS with conventional IAVs^5^, and therefore reassortment events between bat influenza and AIV are not possible^5,34^. The PS are located at the 5′ and 3′ termini of each IAV genomic segment and are responsible for correct packaging of RNA segments by interacting amongst themselves. Juozapaitis et al. have shown that the H17N10 backbone allows the construction of chimeric viruses with segments encoding the IAV glycoproteins with adapted H17N10-specific packaging signals^5,34^. Chimeric H17N10 influenza A viruses carrying the immunogenic AIV glycoproteins hemagglutinin (HA) and neuraminidase (NA) do not reassort with other “non-bat” IAVs.

Based on this knowledge, we created a H17N10 chimeric virus carrying the NA and HA (modified cleavage site from polybasic to monobasic)^35^ genomic segments of H5N1 A/swan/Germany/R65/2006 (R65/06) flanked by H17N10 PS, leading to a 6 + 2 chimeric virus designated as R65_mono_/H17N10. Whether chimeric H17N10 viruses serve as a new efficient type of reassortment-incompetent MLIV in avian or mammalian species was assessed by oronasal prime-boost immunization of chickens of different age and ferrets with the R65_mono_/H17N10 MLIV, and evaluation of the protection level using a homologous HPAIV challenge infection.

## Results

### Vaccination of chickens with R65_mono_/H17N10 protects from lethal challenge with H5N1

**Fig. 1:**
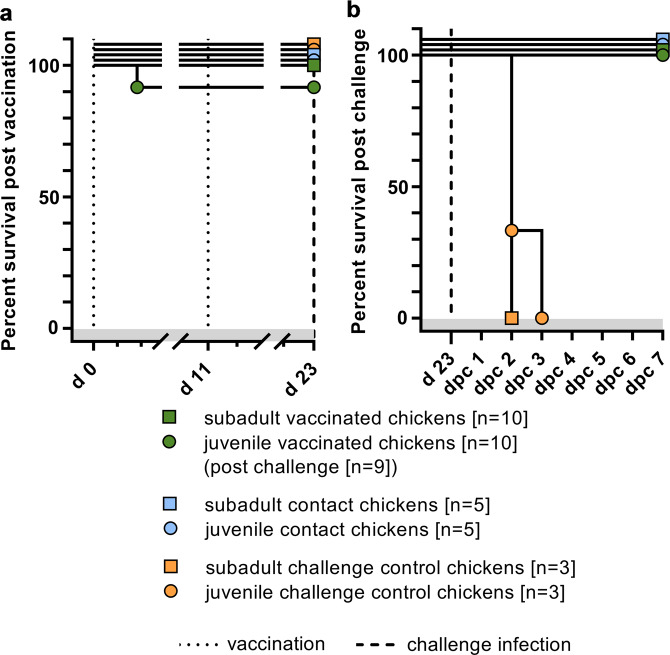
Percentage survival following R65_mono_/H17N10 immunization and subsequent homologous HPAIV challenge infection in chickens. Groups of 4-weeks-old (subadult group) and 1-day-old (juvenile group) chickens were **a** immunized twice with R65_mono_/H17N10 MLIV candidate and **b** challenged with a homologous HPAIV. Five naive contact controls (sentinels) accompanied each group. Three naive controls served as negative-control and from d23 on as positive control to verify the challenge infection.

To evaluate whether R65_mono_/H17N10 MLIV replicates and can protect chickens from lethal challenge with homologous virus, 4-week-old chickens (subadult group) and 1-day-old chicks (juvenile group) were infected and the health status was monitored using a standard clinical score. Of note, the R65_mono_/H17N10 MLIV used for these studies was passaged several times in chicken eggs and day-old-chicks and thereby acquired three additional mutations in HA (A201E, V273A and G339R) and one in M1 (D156N), which might reflect an adaptation to avian cells. However, after primary immunization (PI) and boost immunization (BI), no clinical signs could be observed in both the subadult and the juvenile group. One animal of the juvenile group died 2 days after PI, for unknown reasons (Fig. 1a). Organ samples of this particular animal tested negative for influenza virus RNA by RT-qPCR, and we therefore considered the death of the animal as independent of the immunization.

R65_mono_/H17N10 did not induce any clinical signs or mortality in subadult and juvenile chickens (one unrelated death) and is therefore considered apathogenic to chickens of different age. In order to analyze the protective effect of the immunization against a homologous HPAIV H5N1 challenge infection (CI), clinical score and survival were monitored following CI. Although all naive challenge control chickens succumbed to the CI within 3 days, all vaccinated as well as the naive sentinel animals survived and did not exhibit any clinical signs (Fig. 1b). Thus R65_mono_/H17N10 protected young and subadult chickens against clinical signs and mortality induced by homologous HPAIV H5N1.

### Vaccination of ferrets with R65_mono_/H17N10 protects from lethal challenge with H5N1

**Fig. 2:**
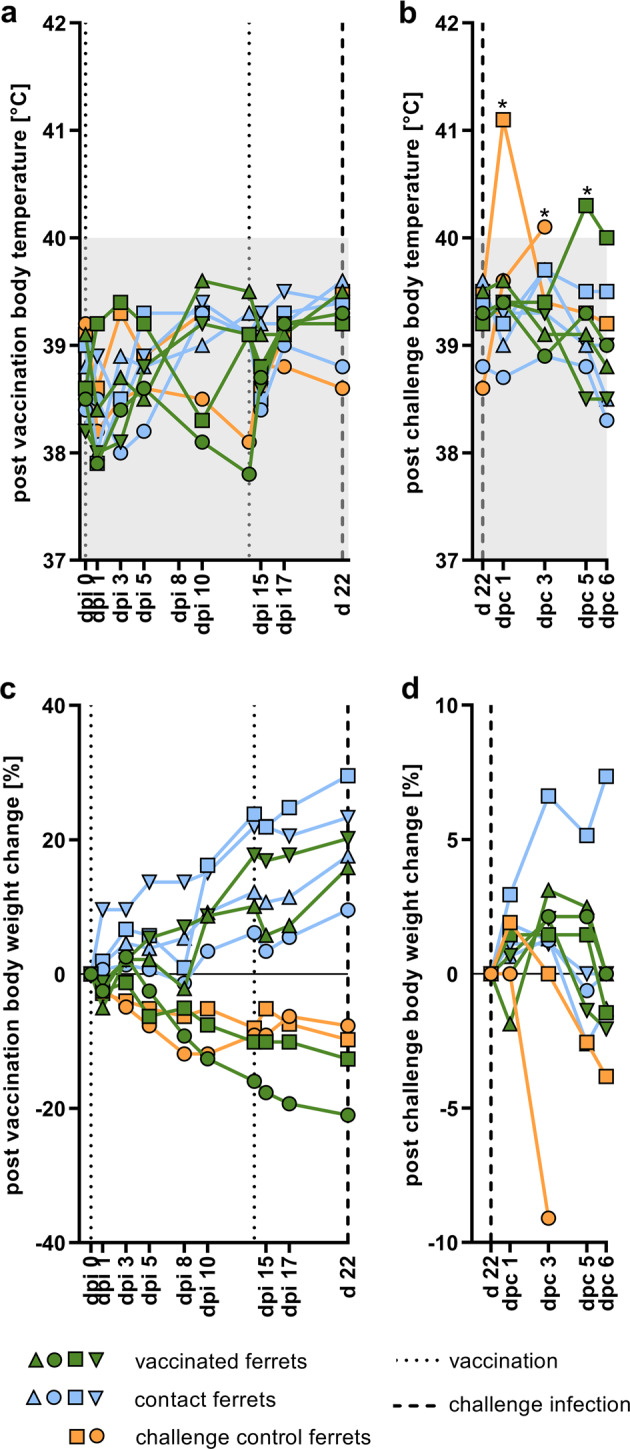
Monitoring of ferret weight and body temperature following R65_mono_/H17N10 immunization and subsequent homologous HPAIV challenge infection. Rectal body temperature (normal range indicated with gray background—asterisks indicate elevated values) and relative body weight change following prime-boost vaccination (**a**, **c**), as well as following challenge infection (**b**, **d**).

The immunization using R65_mono_/H17N10 in ferrets did not induce any clinical signs or increased body temperature (Fig. 2a). After challenge infection, the immunized ferrets did not exhibit any clinical signs, whereas one ferret showed slightly increased body temperatures at dpc5, which decreased the next day (Fig. 2b). Two out of four immunized ferrets lost weight following PI, however, the naive control animals (naive until d22, later served as positive controls for CI) also constantly lost weight following PI (Fig. 2c). Following challenge infection two immunized animals had a minimal weight loss at dpc6 (−2.1 and −1.4% of pre-challenge weight, Fig. 2d). In contrast, the naive positive controls showed higher weight loss (−9.1 and −3.8% of pre-challenge weight) and increased body temperature in response to the challenge infection (Fig. 2b, d). The female positive control ferret displayed lethargy and paralysis of the hind legs at dpc3 and therefore reached the human endpoint criteria.

Thus, the R65_mono_/H17N10 immunization of ferrets did not result in any clinical signs or increased body temperature, while complete protection against clinical signs following CI was induced. Neither immunization nor CI affected the sentinel animals concerning clinical signs, increased body temperatures or body weight.

### Vaccination of chickens with R65_mono_/H17N10 induces sterile immunity against H5N1

**Fig. 3:**
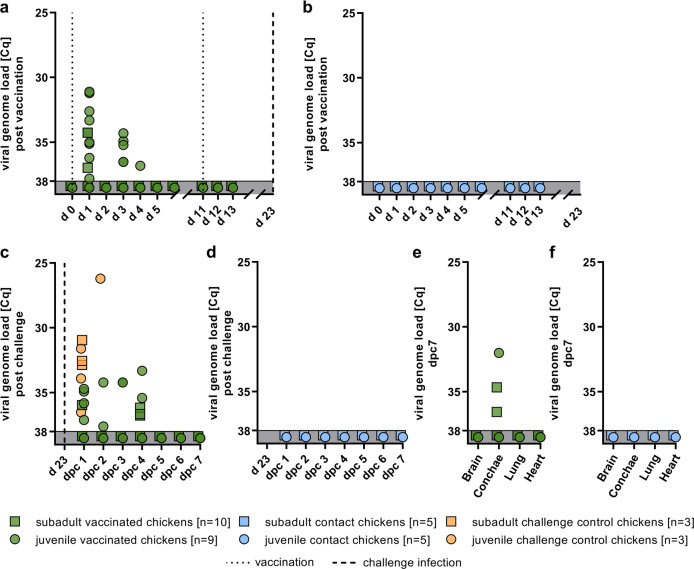
Viral genome loads in swab and organ samples following immunization with R65_mono_/H17N10 MLIV and subsequent homologous HPAIV challenge infection. We examined oropharyngeal swab samples in vaccinated chicken (**a**) and corresponding co-housing contacts (**b**) after prime-boost vaccination and after challenge infection (**c**, **d**). Organ samples were taken post-mortem on dpc7 (**e**, **f**). All samples were examined using pan-influenza PB1-sequence-specific RT-qPCR.

In order to assess the replicative potential of the vaccine candidate R65_mono_/H17N10 in chickens, we sampled oropharyngeal swabs after PI and BI. Swab samples from the first day post PI (d1) showed that 8 of 9 swabs from juvenile chicken were positive for viral genome, furthermore in 3 out of 10 swabs from subadult chicken vaccine virus RNA was detected (Fig. 3a). Although oropharyngeal samples from subadult chicken did not score positive for viral genome, samples from vaccinated chicks displayed viral RNA in five samples on d3, and one sample on d4. Following the BI (d11), none of the samples tested positive. Likewise, none of the direct contact animals were tested positive (Fig. 3b), suggesting that transmission of the R65_mono_/H17N10 to the naive contact animals did not take place. In summary, the viral RNA detection rate of the live vaccine was higher and more durable in the juvenile than in the subadult group, and was restricted to the very first days following PI.

To evaluate subclinical shedding of wild-type challenge virus through vaccinated animals, oropharyngeal swabs were analyzed daily. One day after homologous CI (dpc1), 4 out of 9 juveniles as well as one animal in the subadult group exhibited very weak-positive Cq values (>34.7) from swab samples. However, the naive chickens serving as positive controls demonstrated slightly higher viral RNA loads than vaccinated individuals did (Fig. 3c). On dpc2, only 2 out of 9 juveniles showed very weak-positive Cq values (34.2 and 37.7), whereas all others, including the sentinels (Fig. 3d), scored negative. At that time, one positive control animal was still alive, and the corresponding swab sample tested strongly positive (Cq 26.2; Fig. 3c orange dot at dpc2). Three and four days post CI, individual animals of both groups displayed weak-positive RNA loads (>33.3) until all samples tested negative from 5 dpc onward (Fig. 3c). Viral organ loads following CI can indicate ongoing but unrecognized infections. Animals were killed dpc7, and conchae tissue, brain, heart, and lung samples were taken individually. The samples from brain, heart, and lung were negative from all animals in both groups (Fig. 3e, f). Conchae samples exhibited viral RNA in two samples from subadult chicken, and in one sample from the juvenile group (Fig. 3e). In order to distinguish between PCR-positive but non-infectious samples and samples exhibiting infectious challenge virus, all PCR-positive swab and organ samples collected following CI underwent a cultivation attempt using SPF eggs. Virus recovery was successful only from samples derived from chickens of the positive control group, whereas samples from vaccinated chicken were non-infectious. In summary, these results show that no HPAIV infection established in the vaccinated chickens of both age classes.

### Vaccination of ferrets with R65_mono_/H17N10 prevents from HPAIV H5N1 challenge infection

**Fig. 4:**
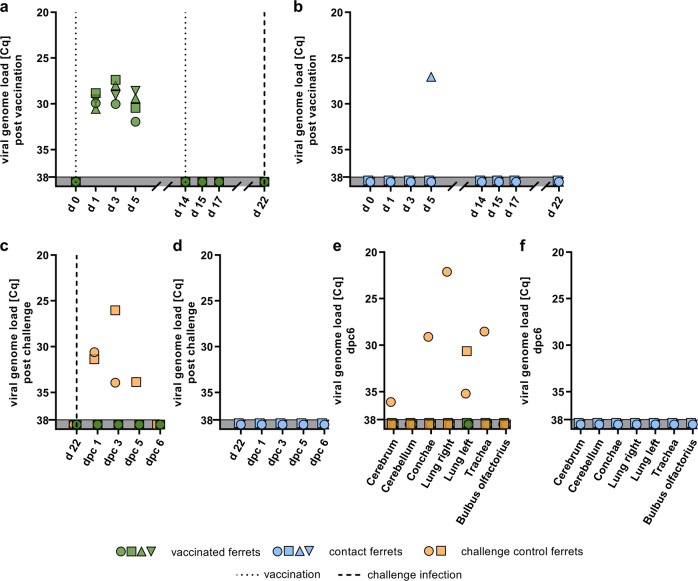
Viral genome loads in ferret nasal washing and organ samples, following immunization with R65_mono_/H17N10 MLIV, and subsequent homologous HPAIV challenge infection (CI). Nasal washing samples were taken following prime-boost vaccination (**a**, **b**) and following the challenge infection (**c**, **d**). Organ samples (**e**, **f**) were taken post-mortem on dpc6 (positive control animal killed on dpc3). All samples were examined using pan-influenza PB1-sequence-specific RT-qPCR.

Following PI, nasal washing samples from immunized ferrets were RT-qPCR positive, at least until d5 (Fig. 4a). In addition, nasal fluid samples from one naive contact sentinel ferret scored positive for viral RNA at d5 (Fig. 4b). No indication of viral secretion was found post BI, already indicating presence of a protecting immunity (Fig. 4a). As a result of the CI, both control animals were positive until dpc5, and one had to be killed at dpc3, whereas samples from all vaccinated animals (Fig. 4c) scored negative. In addition, all contact animals scored negative (Fig. 4d). The examination of several respiratory and neuronal organ samples from the necropsy 6 days post CI revealed no positive tissues from both immunized and sentinel animals (Fig. 4e, f). In contrast, cerebrum, conchae, lung, and trachea tissue sampled from the killed positive control animal tested HPAIV positive (Fig. 4e). In addition, the lung tissue sample of the second positive control animal scored positive with a Cq value of 30.6 at dpc6 (Fig. 4e). In summary, shedding, replication, and systemic infection of HPAIV following CI was prevented successfully by immunization with vaccine candidate R65_mono_/H17N10 in ferrets.

### Vaccination induced low levels of humoral immune response in chickens, but prevented sentinel animals from seroconversion after H5N1 challenge infection of vaccinated chickens

**Fig. 5:**
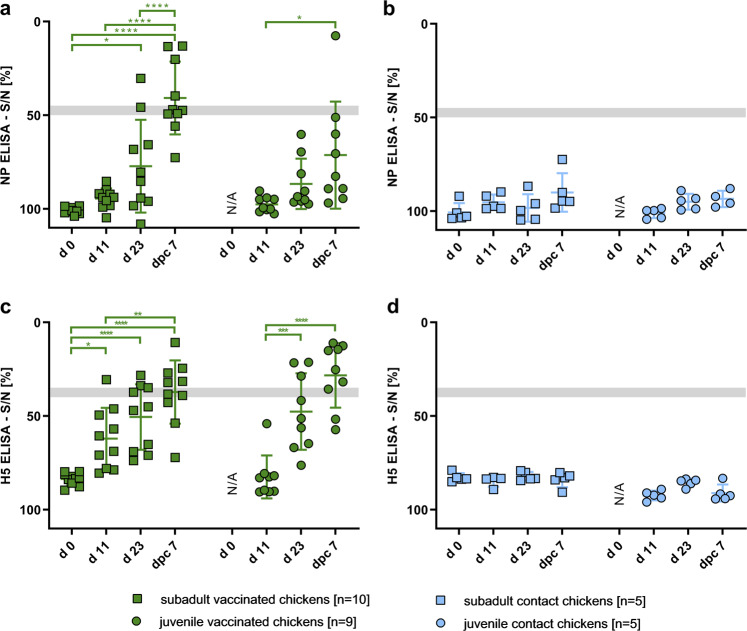
Humoral immune response to R65_mono_/H17N10 immunization and subsequent challenge infection in chickens. Serum samples were taken from all animals on d0, d11, d23, and 7 days post challenge (dpc7 = d30), and evaluated using a competitive Enzyme-linked Immunosorbent Assay (ELISA) specific for nucleoprotein (NP) (**a**, **b**) or hemagglutinin H5 binding antibodies (**c**, **d**). Reduction of the measured signal-to-noise (S/N) % value of a sample indicates presence of specific Ab. Gray bar indicates the reactive response, whereas lower values specify seropositivity. Asterisks indicate the statistical differences between different time points on group level, calculated using one-way ANOVA, and followed by post-hoc Tukey’s test. *p ≤ 0.05, **p ≤ 0.01, ***p ≤ 0.001, ****p ≤ 0.0001. Error bars indicate standard error of the mean (SEM).

Blood samples were collected from the birds in each experimental group on d0 (exclusive day-old-chicks), d11, d23, and d30, serum was separated, and the NP and HA antibody titers were determined by ELISA (Fig. 5). NP-specific Ab levels of the subadult group (Fig. 5a) did not reach seropositivity following PI, and 12 days post BI, only one animal seroconverted. Nevertheless, there was a significant increase in the average S/N level (*p-*value 0.0108) in comparison to the initial values after BI in the subadult group. Highest values were achieved testing subadult chickens after CI. Results detected from individuals of the juvenile group were as follows: none of the chicks seroconverted testing against NP after PI and BI (d11 and d23). After CI, only one juvenile animal seroconverted (d30). All direct contact sentinel animals stayed seronegative for NP-specific antibodies (Fig. 5b) throughout the course of the experiment even after direct contact to vaccinated and challenged animals.

H5-specific antibody titers raised significantly (*p*-value 0.0123) after the PI of the subadult group, as shown in Fig. 5c. This was more evident after BI (*p*-value < 0.0001); however, the increase between prime and boost H5-antibody levels was not significant. Furthermore, in the juvenile animal group, the BI led to significantly (*p*-value 0.0005) increased antibody titers in comparison to levels achieved after PI. Sera from naive juveniles before PI, at an age of 1 day, were not taken for animal welfare reasons. However, as hatched from SPF eggs the chicks were considered seronegative. Although the CI boosted the H5-specific antibody levels in both subadult and juvenile chickens (Fig. 5c, values dpc7), there was a non-significant difference in comparison to antibody levels after boost immunization.

In addition to the missing NP-specific humoral immune response, none of the sentinel animals showed any detectable H5-specific Ab at any time point (Fig. 5d).

By comparing the H5-specific and the NP-specific antibody responses, it became evident that the H5-specific reactions occurred earlier and more prominent than the NP-specific Ab response. In conclusion, R65_mono_/H17N10 induced a moderate level of H5- and very low NP-specific Ab levels.

As shown in Table 1, most animals showed positive hemagglutination inhibition (HI)-titers following the two immunization steps, only two animals of the juvenile group tested positive in the virus neutralization (VN)-test.

**Table 1:**
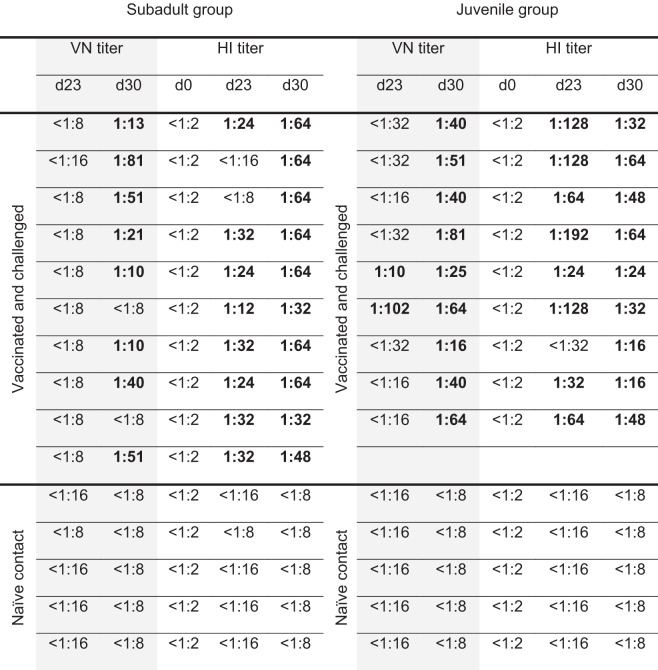
Virus neutralization (VN) test and hemagglutination inhibition (HI) test of chicken sera using homologous H5N1 R65 HPAIV virus.

Shown are the VN- and HI-titers (gray and white background, respectively). Positive tested sera are marked in bold, whereas if tested negative, the lowest tested dilution is indicated.

d23 = 12 days post boost and pre-challenge, d30 = 7 days post challenge.

However, after challenge with HPAIV, neutralizing antibodies were observed in nearly all animals, except for two of the subadult group. The high efficacy of the induced immunity was confirmed by a negative serology of the direct contact controls of both groups also in the VN and HI assays, even 7 days post challenge infection. The sera were also tested against a recent HPAIV H5N8 isolate (tufted duck Germany 2016)^36^ consisting of an phylogenetically different H5 molecule and a heterologous NA subtype, and before challenge infection no cross-reactivity could be detected (Supplementary Table 1). In addition, even sera collected after challenge infection exhibited only minor reactions: one out of 10 (subadult group) and two out of nine (juvenile group) showing low titer reactions (Supplementary Table 1).

Most importantly, the sentinels reacted seronegative 7 days after CI of the vaccinated animals in direct contact, demonstrating the successful prevention of any transmission events after challenge infection. Therefore, R65_mono_/H17N10 administered via the mucosal route in a prime-boost approach induced protective immunity in chickens.

### Vaccine virus induced a fast and pronounced humoral immune response in ferrets

**Fig. 6:**
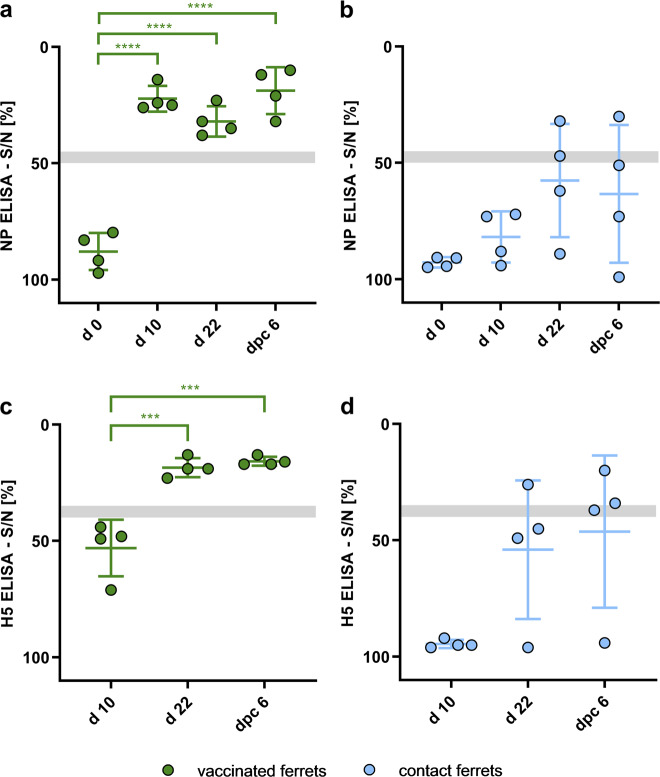
Humoral immune response to R65_mono_/H17N10 immunization and subsequent challenge infection in ferrets. Serum samples were taken from all animals on d0, d10, d22, and 6 days post challenge (dpc6 = d28), and evaluated by a competitive Enzyme-linked Immunosorbent Assay (ELISA) specific for nucleoprotein (NP) (**a**, **b**) or hemagglutinin H5 binding antibodies (**c**, **d**). Reduction of the measured signal-to-noise (S/N) % value of a sample indicates presence of specific Ab. Gray bar indicates reactive response, whereas lower values specify seropositivity. Asterisks indicate the statistical differences between different time points on group level, calculated using one-way ANOVA, and followed by post-hoc Tukey´s test. *p ≤ 0.05, **p ≤ 0.01, ***p ≤ 0.001, ****p ≤ 0.0001. Error bars indicate standard error of the mean (SEM).

In contrast to the experiments with chicken, ferrets of a broad age range (8.4 months–4.6 years) were immunized to analyze both the safety and efficacy of the vaccine prototype in naive, but fully immunocompetent animals. Ferrets were confirmed to be seronegative for IAV antibodies at d0, using a NP-specific ELISA test (Fig. 6a, b). Ten days after PI (d10), all vaccinated ferrets displayed a statistically significant (*p-*value < 0.0001) seroconversion (Fig. 6a). The level of NP-specific Ab remained stable also 12 days after BI (d22) and 6 days after CI (dpc6). Interestingly, one sentinel ferret tested seropositive at d22 and the following time points, whereas for the other two, the S/N value noticeable decrease (Fig. 6b). The H5-response of vaccinated ferrets was significantly boosted (*p-*value 0.0003) following BI, leading to seroconversion on d22 (Fig. 6c). The CI did not further affect the H5-specific antibody levels of the vaccinated animals. Although all sentinels were still seronegative 10 days after PI, one animal was tested positive animal 12 days after BI (d22), and two contact ferrets were positive 6 days post CI (dpc6). The H5-specific antibody response of the ferrets compared with the NP-specific response exhibited a delayed phenotype. These results indicate that R65_mono_/H17N10 is able to induce a pronounced NP-specific immune response ten days after the first mucosal immunization in ferrets, whereas the H5-specific response was delayed reaching comparable levels only following boost immunization (Fig. 6c). Transmission of the R65_mono_/H17N10 vaccine virus from vaccinated to sentinel animals was demonstrated by the fact that one sentinel was seropositive for NP- and H5-Ab 8 days after BI (d22) (Fig. 6b, d).

Twelve days after booster immunization, neutralizing Ab could be detected in almost all immunized ferrets, as shown in Table 2. The titers were just minimally increased following challenge infection, whereas for some animals the titers even decreased. Three out of four naive direct contact animals already showed low VN- and HI-titers before challenge infection, indicating some early transmission events from vaccinated contact animals. The fourth contact animal remained seronegative even after challenge infection. All sera were negative in the VN- und HI-tests against the heterologous tufted duck Germany 2016 HPAIV H5N8 strain (Supplementary Table 2).

**Table 2:** Virus neutralization (VN) test and hemagglutination inhibition (HI) test of ferret sera using homologous H5N1 R65 HPAIV virus.

	VN titer		HI titer		
	d22	d28	d0	d22	d28
Vaccinated and challenged	<1:256	**1:645**	<1:2	**1:770**	**1:24**
	**1:813**	**1:645**	<1:2	**1:96**	**1:48**
	**1:406**	**1:323**	<1:2	**1:32**	**1:32**
	**1:203**	**1:812**	<1:2	**1:64**	**1:32**
Naive contact	**1:32**	**1:102**	<1:2	1:8	1:8
	**1:21**	**1:64**	<1:2	**1:16**	1:8
	**1:512**	**1:512**	<1:2	**1:32**	**1:64**
	<1:64	<1:8	<1:2	<1:64	<1:8

Positive tested sera are marked in bold, whereas if tested negative, the lowest tested dilution is indicated.

d22 = 12 days post boost and pre-challenge, d28 = 6 days post challenge.

The serological results from the ferret immunization experiment indicate that the R65_mono_/H17N10 prototype vaccine virus induced a pronounced immune response with neutralizing Ab. The following challenge infection had only a neglectable impact on the VN- and HI-titers. Nevertheless, the R65_mono_/H17N10 vaccine virus seemingly was transmitted to naive direct contact animals.

## Discussion

In this study, we successfully tested a new type of reassortment-incompetent MLIV (riMLIV). Following non-invasive oronasal application, this vaccine prototype induced protective immunity against homologous HPAIV infection in chickens of different age classes as well as in ferrets. Our vaccine virus candidate is composed of an H17N10 bat influenza-based genetic backbone (PB2, PB1, PA, NP, NS, M) carrying the immunogenic glycoproteins HA (modified to monobasic cleavage site)^35^ and NA from an H5N1 AIV (A/swan/Germany/R65/06) with bat-adapted PS, located at the 3′- and 5′-segment ends. Owing to the incompatibility of the H17N10 PS with conventional IAVs^5,34^, the risk of reassortment with wild-type viruses, the main problem of conventional influenza live vaccine approaches, is neglectable. Therefore, our new riMLIV methodology is highly efficient omitting the risk of introduction of H5 (or H7) genomic segments into circulating viruses^30,37^.

None of the vaccinated chickens as well as the direct contact animals, independently of the evaluated age classes, showed clinical signs following immunization or homologous challenge infection. The demonstration of apathogenicity is of eminent relevance for live vaccines especially in young animals^25^. Just recently, the poor adaptation of the H17N10 backbone, here with H9N2 glycoproteins, to the avian system was shown^38^. This underpins our results of no transmission to direct contact chickens. Generally, restoration of pathogenicity of the attenuated vaccine strain back to the pathogenic phenotype is an important distress of MLIV^39^. Shown by the shedding data and confirmed by constantly seronegative direct contact animals, the R65_mono_/H17N10 strain could be designated as strongly attenuated in chickens already, making further transmission respective restoration of pathogenicity highly unlikely.

In conclusion, the riMLIV complies with three of the recommended features of an AI vaccine to be licensed^40^: (a) to be safe; (b) to be efficacious and (c) to be easy to administer.

In addition to chicken as target population of the tested vaccine prototype, experimental inoculation of ferrets was performed. Ferrets are believed to be the most suitable animal model for studying pathogenicity and transmissibility of influenza infection^33^. Therefore, safety and efficacy of the riMLIV prototype in this suitable mammalian species was evaluated. The apathogenicity of R65_mono_/H17N10 based on the lack of clinical signs or elevated body temperature was also documented for ferrets following a prime-boost immunization scheme with a short interval. Contrasting the results from chickens, the riMLIV in ferrets was transmitted to direct contact animals. Further safety mechanisms, like the generation of cold-adapted mutants^41,42^ and/or the introduction of species-specific siRNA targets^22,43^, could be used to improve the applicability of the prototype as a vaccine for mammalian species.

Interestingly, vaccine virus genome was recorded in nasal washing fluid of one naive direct contact sentinel, and that particular animal also seroconverted at a later time point. In addition, three out of four sentinels tested positive for neutralizing Ab before challenge infection. Therefore, replication and transmission of the vaccine virus prototype from the ferret upper respiratory tract is obviously possible. These results indicate that the chimeric strain R65_mono_/H17N10 is, probably due to the mammalian bat influenza backbone, still more efficient replicating in mammalian than in avian animals. However, after challenge infection none of the nasal washing samples of the vaccinated ferrets tested positive nor did any of the organ samples taken at dpc6. The prime-boost application of riMLIV to ferrets therefore also induced a sterile immunity against a homologous challenge infection, and a good immunity could be expected even after a single shot application, while cross-protection potential does not seem to be exceptional, as shown for H5N8 by in vitro cross-reactivity studies.

The analysis of serum samples using commercial ELISA systems, one detecting antibodies specific for the (avian origin) H5 protein as well as one assay detecting antibodies directed against conserved epitopes of NP, was in principle feasible. Nevertheless, by comparing the ELISA data to the results from VN- and HI-test it becomes clear, that some sera appearing below threshold in the ELISA are positive in the VN- and/or HI-test. These differences might be caused by a reduced overall sensitivity of the NP-ELISA for the distantly related NP protein of H17N10, or a limited replication of the chimeric virus in chicken resulting in the induction of higher levels of HA- than NP-specific antibodies. The latter is supported by the stronger NP-reaction in the ferret experiment where the more efficient replication induced a more pronounced NP-specific response.

Interestingly, the ELISA-based evaluation of the chickens revealed only a moderate antibody response to R65_mono_/H17N10 vaccination in chickens. A likely explanation is the reduced replication of the H17N10 backbone within the avian system. Although the vaccine was passaged several times in embryonated chicken eggs and day-old-chicks, the adaptation of the virus to avian cells is still limited as also demonstrated by the minor oropharyngeal secretion. Thus, serological responses appear less well pronounced. In contrast, the serological response in ferrets was prominent, with high levels of NP-specific and increased H5-specific Ab in the aftermath of PI. The consequent interpretation refers to the mammalian adapted H17N10 backbone that confers replicating capacity to the vaccine virus in ferret tissue.

Regarding the fact that two subadult chickens and one juvenile chicken did not exhibit neutralizing Ab before challenge infection and that only one of the two subadults showed up in the questionable area of the H5-ELISA, while they all survived the challenge infection, raises the question which mechanism leads to protective immunity. It is known that live influenza vaccines can induce protection by induction of mucosal IgA after local application, and CD4^+^ and CD8^+^ T cell responses^44^. Furthermore, T cell subsets and IgA, IgM, and IgG have been shown to be located at the mucosal surface within the nasal-associated lymphoid tissue (NALT) of chickens^45^. Therefore, the level of protection cannot directly be correlated to the humoral antibody titers^17,46^ after immunization with modified live virus vaccines. This is in contrast to IIVs, where protection was mediated by the humoral response only^17,47^. In addition, day-old chicks do not produce sufficient amounts of antibodies until the age of ~7 days^26,27^. The sterile protection in chicks resulting from the riMLIV prototype is particularly impressive knowing these limitations. In this context, further investigations on non-humoral immunity are necessary. Finally, we cannot formerly exclude that antiviral immune responses contributed to the protective effect of vaccination, however, the challenge infection here was always several weeks after the first vaccination and several days after booster immunization. Such innate immune responses are rather short-lived antiviral countermeasures of the host, which might not act at later time points.

Moreover, future research will focus on in ovo single shot immunization using the riMILV. Such a strategy would be especially suitable for vaccination against HPAIV in an endemic situation, allowing the induction of protection as early as possible. One further optimizing aspect would be the opportunity to serologically differentiate infected from vaccinated animals (DIVA)^48^. Development of an ELISA system, which exclusively detects bat influenza virus NP antibodies, but not antibodies specific for the ordinary AIV, would cover this aspect^48–51^.

In summary, we successfully tested a new type of bat flu-based MLIV backbone, carrying the HA and NA of an H5N1 virus, designated as R65_mono_/H17N10 in chickens and ferrets. Both species were completely protected after mucosal immunization and did not spread the challenge virus to naive contact animals. The highly beneficial feature of this MLIV is the absence of any reassortment risk due to the incompatible PS. R65_mono_/H17N10 is apathogenic for ferrets and even day-old chicks. Future work will adapt and evaluate this approach for in ovo application regimes and against further relevant AIV subtypes including highly pathogenic H7 viruses.

## Methods

### Construction of the chimeric vaccine virus prototype

#### Plasmid constructions

PHW2000 plasmids to support the generation of chimeric vaccine virus prototype were the following: the established^5^ A/little yellow-shouldered bat/Guatemala/153/2009 (H17N10) internal segments (GenBank accession no. CY103877, CY103880, CY103874, CY103875 (PA_S550R_), CY103873, and CY103879 (M1_D156N_, M2_N31S; T70A_)) in combination with pHW2000 plasmids coding for A/swan/Germany/R65/06 (H5N1) HA with monobasic cleavage site^35^ and NA (GenBank accession no. DQ464354 (HA_A201E; V273A; G339R and RRRKK351T_) and DQ464355). To make the AIV HA and the NA segments compatible to the H17N10-based backbone, the non-coding region (NCR) of the monobasic HA/R65 segment was replaced with nucleotides 1–131 and 1621–1782 of the bat segment 4 sequence. For generation of the NA rescue vector, the NCRs of the R65 NA segment 6 were replaced with nucleotides 1–122 and 1254–1388 of the bat influenza segment 6 sequence. In addition, ATG codon in the coding sequence of the newly inserted batflu packaging sequences of the HA and NA ORFs were mutated to ACG to prevent initiation of translation at these sites.

#### Virus rescue

The recombinant bat chimera vaccine prototype virus (designated as R65_mono_/H17N10) was generated by the eight plasmids reverse-genetics system^52^. Reverse-genetic virus generation was conducted in 6-well tissue plates, with 10^6^ HEK293T cells (ATTC; CRL-3216) per well, using the eight pHW2000 plasmids (300 ng of each) expressing the individual segments and Lipofectamine 2000 (Invitrogen, Carlsbad CA, USA) according to the manufacturer’s instructions. Eight hours later, the DNA transfection mixture was replaced by infection medium (Dulbecco’s modified Eagle’s medium + 1% penicillin/streptomycin + 0.2% bovine serum albumin + 1% glutamine). After 30 h, TPCK-trypsin was added in the infection medium (0.2 μL/mL). After 48 h, the generated virus (the supernatant) was harvested. The rescued recombinant virus was plaque purified on MDCKII cells before again MDCKII cells were infected for propagation of virus stock. Here HEK293T cells and MDCKII cells were maintained in Dulbecco’s modified Eagle’s medium (DMEM) supplemented with 10% fetal calf serum (FCS), 100 U Penicillin and 100 mg Streptomycin per mL at 37 °C and 5% CO_2_.

Afterwards in order to enable sufficient local replication in avian cells, R65_mono_/H17N10 was passaged several times. In detail, the vaccine prototype resulted from passaging ten times in 11-day-old embryonated eggs (for 96 h), followed by re-isolation from conchae tissue from a day-old chick and additional two times 11 day-old embryonated egg passages, once in 14 day-old embryonated eggs (for 96 h) and finally five times in 18 day-old embryonated eggs (for 48 h). For the passages in older embryonated eggs (14 and 18 day-old), homogenized conchae tissue was sampled and used for further passaging. Lastly, a vaccine virus stock was generated on MDCKII cell culture (Collection of Cell Lines in Veterinary Medicine CCLV-RIE-1061) using trypsin supplementation.

### Challenge virus

To challenge the immunized animals, we used the well-established homologous German wild bird H5N1 highly pathogenic avian influenza virus (HPAIV) index isolate A/Cygnus cygnus/Germany/R65/2006 (H5N1) (“R65”), applying a lethal dose of 10^6^ TCID_50_/animal intranasally^27^.

### Animals

All animal experiments were conducted in biosafety level 3 containment facilities at the FLI and were carried out in accordance with the German Animal Welfare Act, approved by the Committee on the Ethics of Animal Experiments of the Federal State of Mecklenburg-Western Pomerania (registration and approval number LALLF MV/TSD/7221.3-1-023/16).

#### Chickens

For the experiments, 1-day-old and 4-week-old white leghorn chicken (*Gallus gallus* var. *domesticus)* were used. All animals were in house bred from specific-pathogen-free (SPF) eggs (VALO BioMedia GmbH, Osterholz-Scharmbeck, Germany).

#### Ferrets

Healthy and influenza Ab-negative ferrets (*Mustela putorius furo*) of varying age (8.4 month–4.6 years, mean age 2.7 years) were received from PEI and FLI internal breeding program.

### Experimental setup

#### Clinical score

The animals were observed daily for clinical symptoms and classified according to the OIE guidelines as healthy (0), sick (1), severely sick (2), or dead (3)^53^. Mild symptoms were scored as 0.5. When animals were too sick to eat or drink, they were killed and scored as dead on the next observation day.

#### Chickens

We immunized two groups of chickens of different age (subadult group (*n* = 10) - 4 weeks of age (at d0), juvenile group (*n* = 10) - day-old chicks (at d0)) in a prime-boost-approach using R65_mono_/H17N10. Immunizations were carried out intranasally with a dose of 1 × 10^7.5^ TCID_50_ per individual animal in 100 µL volume. The 50% tissue culture infective dose per mL (TCID_50_ per mL) values were calculated by endpoint dilution in duplicate using MDCKII seeded in 96 well plates (Costar®96 Well Clear TC-Treated Multiple Well Plates, Corning Life Science, Wiesbaden, Germany).

**Fig. 7:**
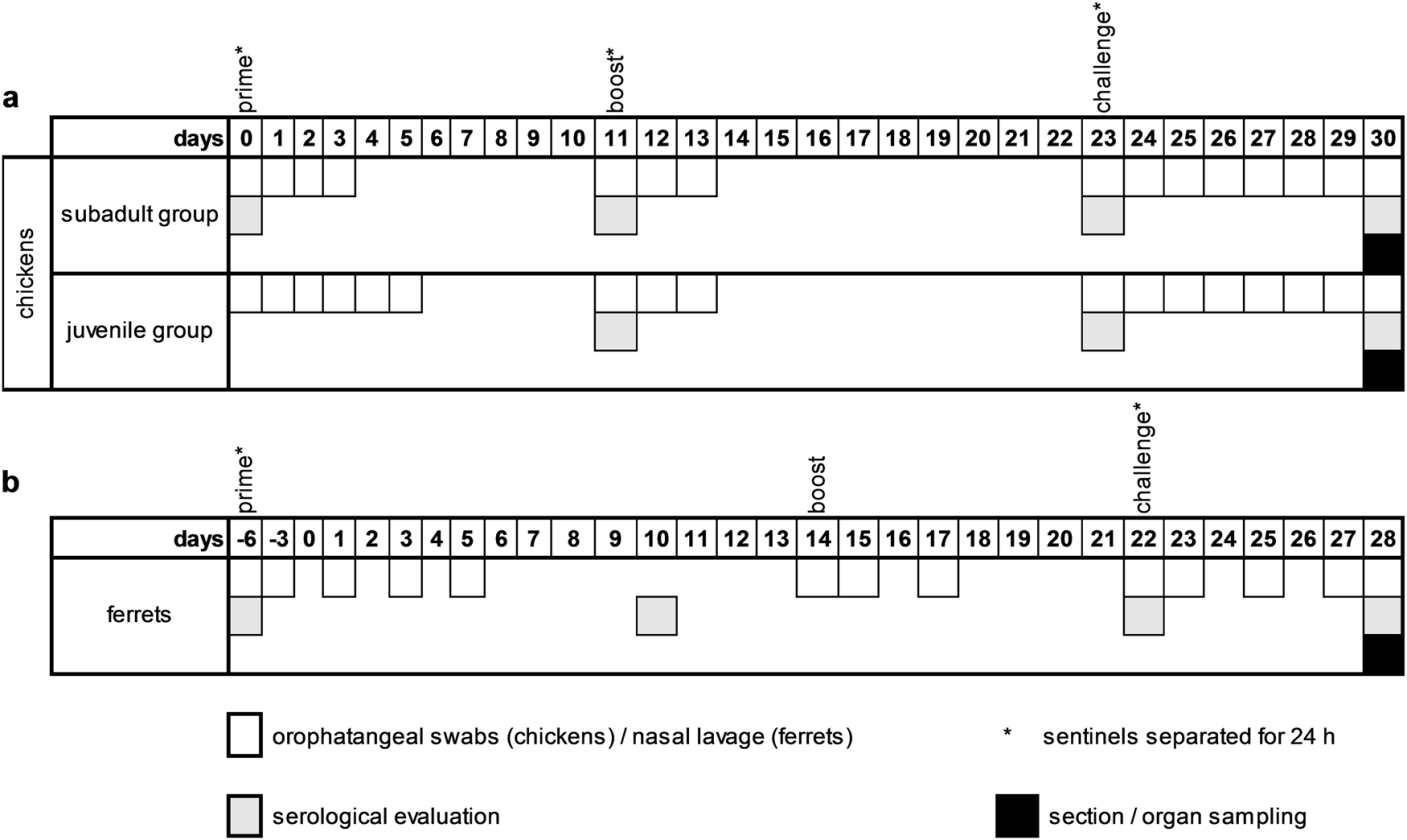
Experimental design. Time frame and sampling regime used for the chicken (**a**) and the ferrets (**b**) vaccination study. Due to animal welfare no blood samples for serological evaluation were taken from chickens of juvenile group on d0.

In each group, five naive direct contact animals (sentinels) of the same age were co-housed with the vaccinated animals. To verify the challenge virus infection, three naive chicken of the same age per group were challenged in parallel. Chickens were monitored daily for morbidity and mortality. Challenge infection was performed intranasally using a lethal dose of 10^6^ TCID_50_ homologous HPAIV per animal. Experimental time frame and sampling regime was as shown in Fig. 7a.

#### Ferrets

Four ferrets were immunized twice with R65_mono_/H17N10 and challenged with homologous HPAIV. Each of the four vaccinated ferrets was co-housed with one naive contact control animal (sentinel). Two naive ferrets served as environment controls throughout the experiment, whereas on d22 they were infected with challenge virus, to verify the challenge infection (CI). Ferrets were monitored daily for morbidity and mortality. Experimental time frame and sampling regime summary is shown in Fig. 7b. Sampling procedure, inoculation as well as weight and rectal temperature measurements were performed on anesthetized ferrets (inhalation anesthesia 5% isoflurane). Immunizations were carried out intranasally with a dose of 1 × 10^7.5^ TCID_50_ per individual animal in 200 µL volume. The 50% tissue culture infective dose per mL (TCID_50_ per mL) values were calculated by endpoint dilution in duplicate using MDCKII seeded in 96 well plates. Challenge infection was performed analogously to the chicken experiment.

## Samples

### Virus replication and shedding

#### Chickens

Virus replication and shedding in chicken were analyzed by taking oropharyngeal swabs (Bakteriette, EM-TE Vertrieb, Hamburg, Germany). Swabs were suspended in 2 mL Dulbecco’s modified Eagle medium supplemented with enrofloxacin 1 mg/mL, lincomycin 1 mg/mL, gentamycin 0.05 mg/mL, and amphotericin 0.05 mg/mL. To check for viral spreading and load in organ tissue, conchae, lung, heart, and brain tissues were examined. Therefore, the organ samples (ca. 8 × 8 mm) were added to 2 mL collection tubes together with 1 mL Dulbecco’s modified Eagle medium (supplemented witch penicillin/streptomycin 0.1 mg/mL (PenStrep®Gibco)) and one stainless steel bead (∅ 5 mm). Subsequently, the organ samples were homogenized using a TissueLyser instrument (Qiagen, Hilden, Germany).

#### Ferrets

Nasal washes were collected from ferrets to measure virus replication by applying 1 mL phosphate-buffered saline (PBS) into each nostril. Organ samples from cerebrum, cerebellum, conchae, lung (left and right), trachea, and bulbus olfractorius were taken, as described for chickens.

### Quantitative real-time RT-PCR

RNA from swab, nasal washing, and organ samples were extracted using the NucleoMag® VETkit (Macherey-Nagel, Düren, Germany) in combination with a Biosprint 96 platform (Qiagen). Viral RNA genome was detected and relative quantified by real-time reverse transcription polymerase chain reaction (real-time RT-qPCR). All RT-qPCR reactions were performed in 12.5 µL volumes using the one-step RT-qPCR Kit qScript™ XLT One-Step RT-qPCR ToughMix® (Quantabio, Beverly, USA) on a CFX96 thermocycler machine (Bio-Rad Laboratories, Hercules, USA). Target sequence for amplification was segment 2 sequence^36^, which detected the bat-IAV-backbone of the chimeric vaccine viruses as well as the HPAIV challenge virus. RT-qPCR Cq values above 38 were considered as negative.

### Virus re-isolation

Viral re-isolation attempts from swab samples following challenge infection of the chickens were done by inoculation of three SPF eggs per swab sample (10 µL/egg). In addition to recording of embryo death on a daily base, harvested allantoic fluid of the inoculated eggs was tested for viral RNA exactly as described. Positive re-isolation was assumed if the Cq value of the AF was lower than that of the initial inoculum.

### Serology

Serum was collected in Monovette (Sarstedt, Nuembrecht, Germany), or Multivette R600®tubes (Sarstedt) from ulnar vein puncture (chickens) or saphenous vein puncture (ferrets). Serum was stored at −80 °C and inactivated at 56 °C for 30 min before usage. For operative realization of HI- and VN-tests, a master plate containing sera dilution series (log^2^ steps) were prepared with PBS, using 1:8 or higher dilution according to the available sample volume.

#### ELISA

Serological responses were determined using competitive Enzyme-linked Immunosorbent Assay (ELISA)-Kits (ID-vet, Montpellier, France) specific for nucleoprotein (NP) or hemagglutinin H5 binding antibodies according to the manufacturer’s instructions using a ELISA reader, Infinite M200PRO (Tecan, Männedorf, Switzerland). S/N reduction indicates presence of specific Ab.

#### HI-test

Hemagglutination activity was determined in microtiter plates by using 1% chicken erythrocytes. The reactions were performed in phosphate-buffered saline (PBS) at room temperature (~20 °C). The hemagglutination inhibition assay was conducted as described in the International Office of Epizootics (OIE) Manual of Diagnostic Tests and Vaccines for Terrestrial Animals^53^ in duplicate. The HI-titer was expressed as the mean value of the highest dilution of serum causing complete inhibition of agglutination of 4 HAU antigen of each duplicate.

#### VN-test

The virus neutralization test (VN) was performed with triplicates of serial dilution prepared in a 50-µL volume of cell culture medium in 96-well plates. The diluted serum samples were mixed with an equal volume of medium containing HPAIV R65/06 10^3.3^ TCID_50_/well. After 1 h of incubation at 37 °C in a 5% CO_2_ humidified atmosphere, 100 µL of MDCK cells at 1.5 × 10^5^ per mL were added to each well. The plates were incubated for 3 days at 37 °C in 5% CO_2_. Viral replication was assessed by visually scoring the cytopathic effect without staining. Each assay was validated by comparison with positive- and negative-control sera from chickens/ferrets and by titration of the virus dilutions used.

### Statistics

To evaluate the statistical significance of ELISA antibody titers among groups one-way ANOVA, followed by post-hoc Tukey’s test for multiple group-by-group comparison using a confidence level of 0.95 (GraphPad Software version 7.04, San Diego, CA, USA) was used. Parenthesis and asterisks represent significant (*p* = 0.01–0.05 (*)), very significant (*p* = 0.001–0.01 (**)) and extremely significant (*p* = 0.0001–0.001 (***) and *p* < 0.0001 (****)) statistical differences. Error bars indicate standard error of the mean (SEM).

### Reporting summary

Further information on research design is available in the Nature Research Reporting Summary linked to this article.

## Supplementary information

Supplementary Information

Reporting Summary

## Data Availability

All data generated or analyzed during this study are included in this published article (and it’s Supplementary Information Files). All relevant data are also available upon request from the authors.
