# Supplementary material for: A modified live bat influenza A virus-based vaccine prototype provides full protection against HPAIV H5N1
Source: NPJ Vaccines. 2020 May 15;5:40. doi: 10.1038/s41541-020-0185-6 (PMC7229168; doi:10.1038/s41541-020-0185-6)
Supplement: Supplementary file 1 — Supplementary Information [file 41541_2020_185_MOESM1_ESM.pdf]

Supplementary information

Supplementary Table 1: Virus neutralization (VN) test and hemagglutination inhibition (HI) test of chicken sera using a heterologous HPAIV H5N8 virus (A/tufted duck/Germany/AR8444-L01987/2016, clade 2.3.4.4 group B). *Shown are the VN- and HI-titers and positive reactions are indicated in bold. d23 = 12 days post boost, d30 = 7 days post challenge.*

		**VN titer**		**HI titer**				**VN titer**		**HI titer**	
		**d23**	**d30**	**d23**	**d30**			**d23**	**d30**	**d23**	**d30**
**Subadult group**	Vaccinated and challenged	<1:8	<1:8	<1:8	<1:8	**Juvenile group**	Vaccinated and challenged	<1:32	**1:10**	<1:32	<1:8
		<1:16	<1:8	<1:16	<1:8			<1:32	<1:8	<1:32	<1:8
		<1:8	<1:8	<1:8	<1:8			<1:16	<1:8	<1:16	<1:8
		<1:8	<1:8	<1:8	<1:8			<1:32	**1:25**	<1:32	<1:8
		<1:8	<1:8	<1:8	<1:8			<1:8	<1:8	<1:8	<1:8
		<1:8	<1:8	<1:8	<1:8			<1:32	**1:10**	<1:32	<1:8
		<1:8	<1:8	<1:8	<1:8			<1:32	<1:8	<1:32	<1:8
		<1:8	<1:8	<1:8	<1:8			<1:16	<1:8	<1:16	<1:8
		<1:8	<1:8	<1:8	<1:8			<1:16	<1:8	<1:16	<1:8
		<1:8	**1:10**	<1:8	<1:8						
	Naïve contact	<1:16	<1:8	<1:16	<1:8		Naïve contact	<1:16	<1:8	<1:16	<1:8
		<1:8	<1:8	<1:8	<1:8			<1:16	<1:8	<1:16	<1:8
		<1:16	<1:8	<1:16	<1:8			<1:16	<1:8	<1:16	<1:8
		<1:16	<1:8	<1:16	<1:8			<1:16	<1:8	<1:16	<1:8
		<1:16	<1:8	<1:16	<1:8			<1:16	<1:8	<1:16	<1:8

Supplementary Table 2: Virus neutralization (VN) test and hemagglutination inhibition (HI) test of ferret sera using a heterologous HPAIV H5N8 virus (A/tufted duck/Germany/AR8444-L01987/2016, clade 2.3.4.4 group B). *Shown are the VN- and HI-titers and positive reactions are indicated in bold. d22 = 12 days post boost, d28 = 6 days post challenge.*

	**VN titer**		**HI titer**	
	d22	d28	d22	d28
Vaccinated and challenged	<1:256	<1:8	<1:256	<1:8
	<1:16	**1:13**	<1:16	<1:8
	<1:16	<1:8	<1:16	<1:8
	<1:64	<1:8	<1:64	<1:8
Naive contact	<1:8	<1:8	<1:8	<1:8
	<1:16	<1:8	<1:16	<1:8
	<1:16	<1:8	<1:16	<1:8
	<1:64	<1:8	<1:64	<1:8
